# The Alimentary Rest Treatment of Diabetes

**Published:** 1916-07-29

**Authors:** 


### THE ALIMENTARY REST TREATMENT OF DIABETES.

### Some Details of a Promising Regimen.

In spite of the labours of a host of physicians for a century or more, the cause of ordinary diabetes mellitus remains as great a mystery as its treatment is unsatisfactory. Though by diet and other means it is possible in most cases to reduce the amount of sugar passed in the urine, and sometimes to abolish it altogether, temporarily, at least, it is nevertheless true that in the majority of cases the progress of the disease tends steadily downwards until directly or indirectly it leads to the death of the patient. The outlook is not by any means of uniform gloominess; in patients under forty, and especially under twenty-five, the fatal issue is seldom avoidable or long postponed, whereas in the middle-aged or elderly diabetes is of much less serious a character, and may exist for many years without appearing to do the patient much harm. But although this rough rule holds good for the bulk of cases, it is by no means invariable. Cases of spontaneous cure in quite young people are on record; and rapidly-fatal cases are not unknown in the aged. No medicinal treatment seems to have any indisputable success in controlling the disorder. In the last stages of coma preceding death it is nearly always possible to revive the patient temporarily by intravenous injections of sodium bicarbonate, and there have been rare instances where this treatment has been followed by recovery from the disease; but then there have also been (equally rare) recoveries from coma without any treatment at all.

### The Failure of Therapy in Diabetes.

In dealing with a disease so uncertain in its course, so common, so deadly, so little amenable to empirical therapeutics, the one real hope of advance lies in discovery of its cause or causes. Nor must it be overlooked that there may be more than one disease—possibly several—characterised by the presence of sugar (dextrose) in the urine, and so far undifferentiated. This variability has led at various times to extravagantly optimistic reports on the merits of many drugs as "cures" for diabetes; and more recently of equally fantastic hopes based on vaccines, enzymes, and so forth. Hitherto, however, a carbohydrate-free diet has been the mainstay of therapeutists, though since this century opened several clinicians (especially in Germany) have broken away from the rigid dietary which was formerly in vogue. Last year F. M. Allen,* an American physician, published an account of a new system of diet, based partly on experimental work on animals into the causation of the disease; and recently a most favourable preliminary report of his treatment has been published in this country by Dr. Leyton,† physician to the London Hospital.

### The F. M. Allen Dietary.

The Allen treatment, which demands some considerable degree of fortitude on the part of the patient and the most careful control and supervision on that of the physician, commences with complete starvation of from two to ten days, until the urine is free from sugar. (It was long ago observed that fasting rapidly reduces the output of sugar in the urine, as well as the thirst and polyuria that are characteristic of diabetes.) The patient is kept in bed, and water is allowed freely during this period of starvation, also one cup of tea and one cup of coffee a day, if desired; on the third and succeeding days 300 c.c. of clear meat broth in divided portions is allowed. If diacetic acid is present in the urine, alcohol is also administered, in a quantity fixed according to the patient's weight.

When a twenty-four hours' sample of urine has been found free from sugar, a small quantity of certain vegetables (under half a pound) containing not more than 5 per cent. of carbohydrates is allowed during the next twenty-four hours. This is gradually increased both in amount and in carbohydrate strength, until fruit, potato, oatmeal, and even bread are being taken. If sugar reappears a fast is at once enjoined until it vanishes, after which the patient starts again at half the quantity of carbohydrate food that was being taken when the sugar appeared, and does not exceed this allowance for a fortnight. The object of this starvation, followed by vegetable diet, is to establish a toleration of carbohydrate foodstuffs, up to 3 grams of carbohydrate per kilogram* of body weight, if possible.

While this tolerance of carbohydrate is being built up a protein tolerance is also aimed at. When the urine has been sugar-free for two days—*i.e.*, on the next day after the vegetable diet is started, three eggs are added to the daily diet, followed by increasing amounts of meat until 1 gram per kilogram of body weight is being taken. If the carbohydrate tolerance is zero, then only $\frac{2}{3}$ gram of protein per kilo. of weight is aimed at. When this full protein tolerance is established fat is added to the diet and increased in the same way until the patient ceases to lose weight.

### A Weekly Fast-day.

In all cases a weekly fast-day is practised. If carbohydrate tolerance is less than 20 grams, the fast is absolute; if between 20 and 50 grams, vegetables containing 5 per cent. carbohydrate and half the usual quantity of protein and fat are allowed; if between 50 and 100 grams, vegetables containing 10 and 15 per cent. of carbohydrate are allowable; if more than 100 grams, then half the usual total of carbohydrate can be taken.

It will be seen that the treatment means a very distinct starvation for what may be few or many days. It is said that a patient who begins this system and then gives it up is worse off than if he had never started it; it is, therefore, important not

* *Boston Medical and Surgical Journal*, February 18, 1915.

† *Practitioner*, July 1916.

* By an unfortunate misprint in Dr. Leyton's paper a kilogram is set down as equal to 22 lb., instead of 2.2 lb. (avoirdupois).

to recommend it except to those who have enough intelligence and self-control to stick it out. Dr. Leyton states that the inevitable loss of weight does not cause any great inconvenience, and that it practically ceases after a fortnight in most cases. In order to minimise discontent, he favours the plan of having several patients under treatment at once in a single ward; thus those in the starvation ("alimentary rest") stages are cheered by the improvement to be seen in those who are further advanced, and by the prospect of making similar progress. He also allows spinach, lettuce, and celery which have been boiled in three changes of water and flavoured with broth, pepper, and salt, in about three times the quantity laid down above; by this means most of the carbohydrate is boiled out of them, and the bulk is sufficient to give the patient the sensation of fulness. He encourages patients to test their own urine and to continue to do so for many months, in order to decide when a fast-day is necessary and to give them ocular demonstration of their improvement or of any relapse. Neither he nor the American physicians claim an infallible cure by this treatment; further details are promised when more data have been accumulated. Meanwhile, it can safely be said that the Allen system of diet is highly promising, and deserves a trial in British hospitals.

